# Supplementary material for: Raman and fluorescence characteristics of resonant inelastic X-ray scattering from doped superconducting cuprates
Source: Sci Rep. 2016 Jan 22;6:19657. doi: 10.1038/srep19657 (PMC4726252; doi:10.1038/srep19657)
Supplement: Supplementary Information [file srep19657-s1.pdf]

Supplementary Information of
"Raman and fluorescence characteristics of resonant inelastic X-ray scattering from doped superconducting cuprates"

H. Y. Huang,[1,2] C. J. Jia,[3] Z. Y. Chen,[4] K. Wohlfeld,[5] B. Moritz,[3] T. P. Devereaux,[3] W. B. Wu,[1] J. Okamoto,[1] W. S. Lee,[3] M. Hashimoto,[3] Y. He,[3,6] Z. X. Shen,[3,6,7] Y. Yoshida,[8] H. Eisaki,[8] C. Y. Mou,[4] C. T. Chen,[1] and D. J. Huang[1,4]

[1]*National Synchrotron Radiation Research Center, Hsinchu 30076, Taiwan*
[2]*Program of Science and Technology of Synchrotron Light Source, National Tsing Hua University, Hsinchu 30013, Taiwan*
[3]*SIMES, SLAC National Accelerator Laboratory, Menlo Park, California 94025, USA*
[4]*Department of Physics, National Tsing Hua University, Hsinchu 30013, Taiwan*
[5]*Institute of Theoretical Physics, Faculty of Physics, University of Warsaw, PL-02093 Warsaw, Poland*
[6]*Department of Applied Physics, Stanford University, Stanford, California 94305, USA*
[7]*Department of Physics, Stanford University, Stanford, California 94305, USA*
[8]*Nanoelectronics Research Institute, National Institute of Advanced Industrial Science and Technology, Tsukuba, Ibaraki 305-8562, Japan*

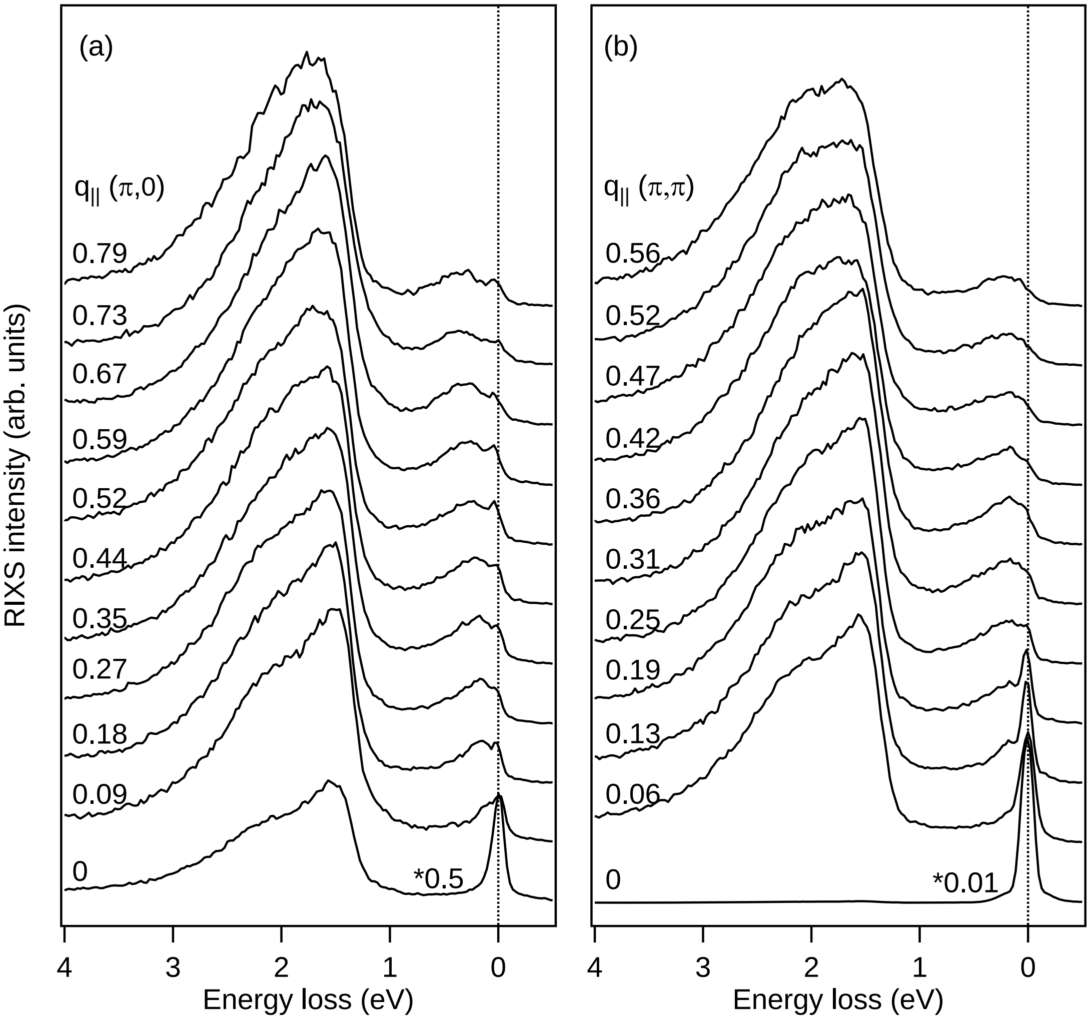

Supplementary Figure 1. Momentum-dependent L_3-edge RIXS spectra along (a) antinodal and (b) nodal directions measured with π-polarized incident X-rays. $\mathbf{q}_{\parallel}$ is expressed as $q_{\parallel}(\pi, 0)$ or $q_{\parallel}(\pi, \pi)$. The incident angle θ_i measured between the incident X-ray and sample surface is varied between 65° at $q_{\parallel} = 0$ to 115° at the largest $q_{\parallel}$. All the data are normalized to the dd excitations.

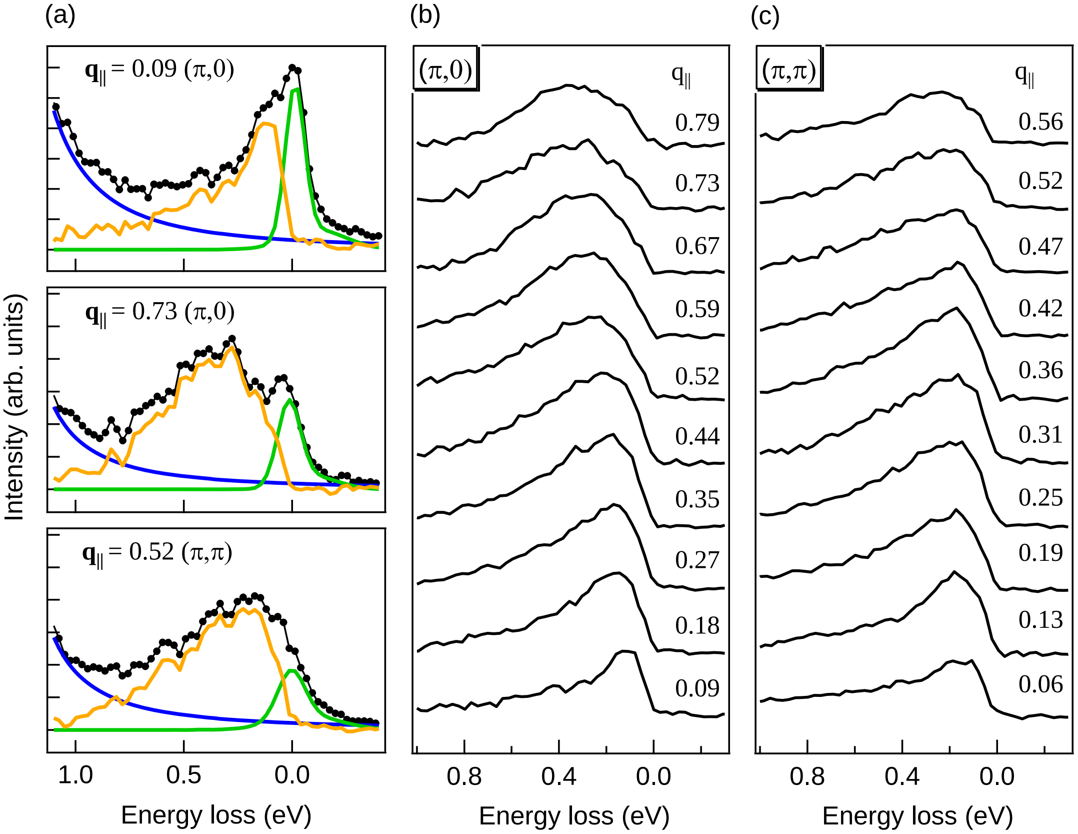

Supplementary Figure 2. Momentum-dependent L_3-edge RIXS spectra after subtraction of elastic peak. (a) Selected original RIXS data plotted together with the elastic line (green) and the background tail of the *dd* excitation (blue) at selected $\mathbf{q}_{\|}$ along the antinodal $(\pi, 0)$ and nodal (π, π) directions. The data after background subtraction are plotted with orange lines. (b) & (c) RIXS spectra of various $\mathbf{q}_{\|}$ after removing the elastic line and the background of *dd* excitations.
